# 

**Published:** 1898-11-19

**Authors:** 


					CADBURY'S *& lOT 1* CADBURY'S
COOOA Y MM OOCOA
ABSOLUTELY PURE. '-???" NO DRUQ8 OR ALKALIES USED.
vr ?ulasaan' hebtbrm Ihei&marx
^ 634. Vol. XXV. LIXSSH SiTTOrAV,
Nov. 19th, 1898.
? ???NOMOLKjuulNORW1CH HOSPLJAJ. '
[dub.oriptionTtonB,] pBICE TWOPENCE.

				

